# A qualitative study exploring the factors influencing maternal healthcare access and utilization among Muslim refugee women resettled in the United States

**DOI:** 10.1371/journal.pone.0307192

**Published:** 2024-08-16

**Authors:** Sarah Yeo, Yoonjung Kim-Hines, John Ehiri, Priscilla Magrath, Crista Johnson-Agbakwu, Kacey Ernst, Sahra Ibrahimi, Halimatou Alaofè

**Affiliations:** 1 The University of Arizona Cancer Center, Tucson, Arizona, United States of America; 2 Education and Language Education Department, International Christian University, Tokyo, Japan; 3 Department of Health Promotion Sciences, Mel and Enid Zuckerman College of Public Health, University of Arizona, Tucson, Arizona, United States of America; 4 Chan Medical School, University of Massachusetts, Worcester, Massachusetts, United States of America; 5 Epidemiology and Biostatistics Department, Mel and Enid Zuckerman College of Public Health, University of Arizona, Tucson, Arizona, United States of America; 6 Department of Global Health, Denison University, Granville, Ohio, United States of America; Access Alliance Multicultural Health and Community Services: Access Alliance, CANADA

## Abstract

Although a large number of Muslim refugees have resettled in the United States for the last decades, few studies have looked into maternal healthcare access and utilization among Muslim refugee women in the country. This qualitative study was conducted to explore the factors influencing maternal healthcare access and utilization among Muslim refugee women resettled in the United States. In-depth interviews were conducted among Afghan, Iraqi, and Syrian refugee women (n = 17) using an interview guide informed by Social Cognitive Theory and its key constructs. The interviews were recorded and transcribed verbatim, imported into MAXQDA 2020 (VERBI Software), and analyzed based on qualitative content analysis. Data analysis revealed several themes at the micro, meso, and macro-levels. Micro-level factors included women’s attitudes toward hospitals and prenatal care, as well as their life skills and language proficiency. Meso-level factors, such as cultural norms and practices, social support and network, as well as health care provider characteristics, were also identified. Macro-level factors, such as the complex healthcare system and access to insurance, also appeared to influence maternal healthcare access and utilization. This study revealed the complex contextual factors that refugee populations face. Given the population’s heterogeneity, a more nuanced understanding of refugee maternal health is required, as are more tailored programs for the most vulnerable groups of refugee women.

## Introduction

Climate change, natural disasters, conflicts, wars, and economic hardship have resulted in an unprecedented number of forcibly displaced people worldwide. Over the last decade, the refugee population around the world has more than doubled, from 15.4 million to 35.2 million [1]. Refugees have poorer health conditions and adverse health outcomes due to limited access to healthcare services, lower levels of health literacy, and economic insecurity [2]. Moreover, they face a substantial disease burden resulting from the hardships and inadequate living conditions encountered during their migration [3]. Refugees frequently encounter psychological stress and mental health challenges due to their experiences and exposures to traumatic events and violence in their home country and on their journey [4].

Overall, refugee and migrant mothers have poorer knowledge of and access to maternal and child health services [2]. According to one meta-analysis conducted in Western Europe, migrant women, including refugee women, are two times more likely to die from pregnancy-related complications than women in the host countries [5]. They also have increased maternity-related risks and adverse maternal outcomes due to high care costs, unfamiliarity with the healthcare system, language barriers, and discrimination [6–9]. Furthermore, refugee women’s use of prenatal care was lowest among migrants [10].

However, access to and utilization of maternal healthcare by refugee women is largely unknown in the United States, one of the few high-income countries with an increasing maternal mortality ratio [11]. Despite all the medical and technological advances and discoveries over the last four decades, pregnancy-related mortality has been increasing in the country since 1987, and the burden falls disproportionately on the marginalized [12, 13]. In the United States, however, data on maternal mortality ratios do not provide disaggregated figures for migrants or refugees. The critical gaps in data and health information systems regarding the health of refugees and migrants prevent a full understanding of this issue [2, 14].

Insufficient focus has been placed on the refugee population hailing from Muslim countries despite a substantial number of refugees being resettled in the United States originating from these regions. From 2011 to 2023, refugees from Muslim-majority countries such as Afghanistan, Iraq, and Syria comprised more than one-third of the total number of resettled refugees in the United States [15]. Some studies have investigated the detrimental effects of political events such as 9/11 and the 2017 Executive Order 13769, commonly referred to as the “Muslim Travel Ban,” which restricted immigration from Muslim-majority nations to the United States, on the maternal health of Muslim immigrant women [16]. Another commentary discussed maternal healthcare challenges faced by Arab Muslim refugee women, identifying different barriers to care such as transportation and access to insurance [17]. However, there remains a dearth of empirical research concerning the specific experiences of maternal healthcare among Muslim refugee women who have resettled in the country. This qualitative study was conducted to explore the factors influencing maternal healthcare access and utilization among Muslim refugee women resettled in the United States.

## Methods

Data were gathered through in-depth interviews using a semi-structured interview guide. The interview guide was initially informed by Social Cognitive Theory and its key constructs [18]. Social Cognitive Theory identifies the primary determinants of health and the process through which knowledge translates into health practices [18]. It provides a framework for understanding the individual and social factors that facilitate or hinder health behaviors. By examining constructs such as self-efficacy, knowledge, outcome expectations, goals, and perceived facilitators and barriers, Social Cognitive Theory helps to elucidate the mechanisms that influence health practices at both the individual and community levels. The questions were intended to explore knowledge and perceptions concerning maternal care, experiences of maternal healthcare services, and perceived barriers and facilitators among refugee women. Examples of the questions can be found elsewhere [19]. After the open-ended questions were developed based on the key constructs of Social Cognitive Theory, it had been pretested with two refugee women, and the interpreters checked the guide and provided feedback as well. Several questions were added based on new themes that emerged from the interviews.

The study involved Muslim refugee women residing in the United States, irrespective of their duration of stay in the country, who had experienced pregnancy or childbirth in the United States. The first author conducted the interviews with a female interpreter between May and December 2022. Most of the interviews were done in the participants’ houses. One interview was done over the phone as the participant had migrated to another state, and the other was conducted online. Following the completion of data collection, the interpreters were interviewed to make sense of the data, particularly themes related to cultures and religions.

### Recruitment

The sample size for participants was determined based on literature that suggested 12–16 participants could be adequate for interviewing relatively homogeneous groups on a specific topic [20, 21]. Refugee resettlement agencies and key informants helped the recruitment process, and the snowball technique was also utilized to recruit women. Initially, only Arabic-speaking refugee women from countries, such as Iraq and Syria, were recruited. However, after the inception of this study, the turmoil in Afghanistan drove more than 120,000 Afghans out of the country, and approximately 76,000 have resettled in the United States since the Taliban takeover of the country. A resettlement agency, one of the community partners that facilitated the recruitment process, proposed to include Afghan refugees as part of this study, and they became part of the study to provide relevant information for the agency.

### Data collection

All interviews were conducted by the first author with the assistance of a female interpreter. All the interpreters were female native speakers who were fluent both in English and Arabic, Dari, or Pashto. Prior to the interview, the interpreters had training on interpretation and orientation concerning the research aims and ethics. They checked whether the interview guide was culturally appropriate and provided some strategies concerning recruitment. Each study participant received $25 cash as compensation for their participation. Participants who could read were given a consent form translated into their native languages and time to read it. If they were illiterate, the interpreter reviewed the consent form with them. The interview began with demographic questions after the consent form was signed. With informed consent, the interview was audio-recorded. Transcriptions were made verbatim only for the remarks by the interviewer and the interpreter. After the English section was transcribed, the interpreters reviewed the recording and transcripts to double-check the accuracy of the interpretation. If any missing information was discovered, they noted it and updated the transcripts. The transcripts were then imported into MAXQDA 2020 (VERBI Software) for analysis.

### Data analysis

**Fig 1:**
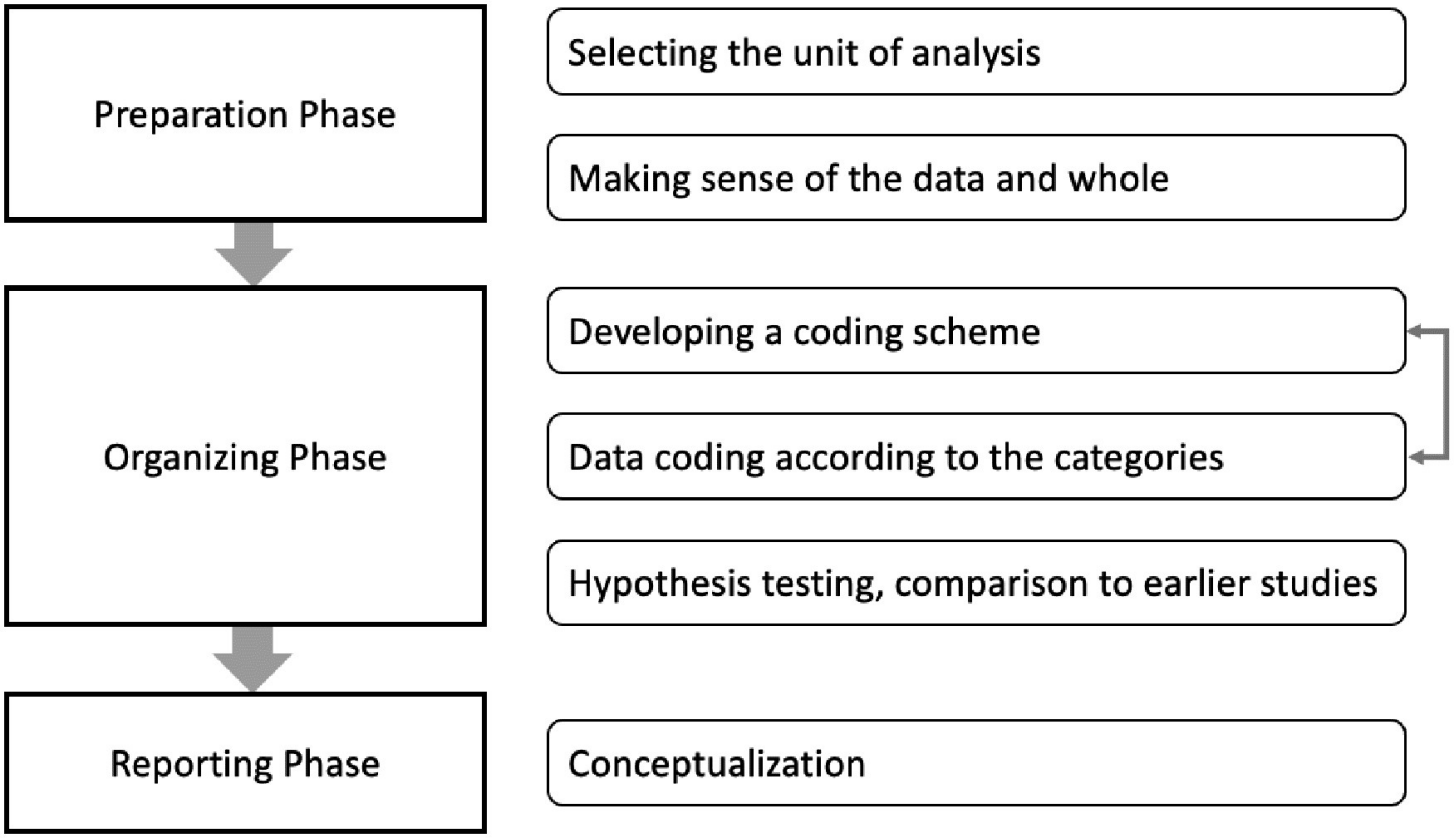
Qualitative content analysis process, adapted from [22].

The qualitative content analysis process [22] guided the analysis process (Fig 1). First, we selected the unit of analysis. The full responses and discussions that fell under one elicitation served as the unit of analysis [23]. The scripts were then read several times to immerse in the data and become acquainted with it. Then, notes that could be used as a code or category were made [22]. As the questions were guided by the Social Cognitive Theory and its constructs, we used deductive content analysis and developed a coding scheme. Emerging codes were also added to the codebook even though they were not part of the theory concepts to fully capture the meanings and nuances of the interviews. Thus, developing the coding scheme and data coding were conducted iteratively (Fig 1).

The first transcripts were reviewed by two researchers (SY and YK), who took notes and discussed potential codes. They coded two identical transcripts after developing a codebook and calculated intercoder agreement (with a minimum code overlapping rate of 90% at the segment level, the default value in MAXQDA). The initial intercoder agreement was 88.8%, and coding discrepancies were investigated and corrected in the codebook. A consensus was reached through the iterative process, and the remaining transcripts were coded. The study was approved by the Institutional Review Board at the University of Arizona (Protocol number: 2104716241).

## Results

### Characteristics of participants

In-depth interviews were conducted with a total of 17 women. Table 1 summarizes the study participants’ characteristics. The average age was 34, with a range of 17 to 52. They were all covered by Medicaid, which provides health insurance to people with low incomes and those who meet other eligibility requirements. The majority of the women interviewed possessed limited English proficiency, regardless of their length of stay in the United States. Three women spoke English fluently, and their interviews were conducted in English. All of the other women were housewives, with the exception of the three women who spoke good English.

**Table 1:** The characteristics of study participants (n = 17).

		n (%)
Age (mean, range)		34 (17–52)
Education level	No education	4 (24)
	Elementary	6 (35)
	Middle school	1 (6)
	High school	2 (12)
	Bachelor’s degree	4 (24)
Employment status	Housewife	14 (82)
	Part-time worker	1 (6)
	Full-time worker	1 (6)
	Student	1 (6)
Health insurance	Yes	17 (100)
	No	0 (0)
Number of children (mean, range)		4 (1–8)
Self-reported English level	Very well	3 (18)
	Well	0 (0)
	Not well	9 (53)
	Not at all	5 (29)
Country of origin	Afghanistan	8 (47)
	Iraq	3 (18)
	Syria	6 (35)
Number of years stayed in the US (mean, range)		4 years (5 months-15 years)

**Fig 2:**
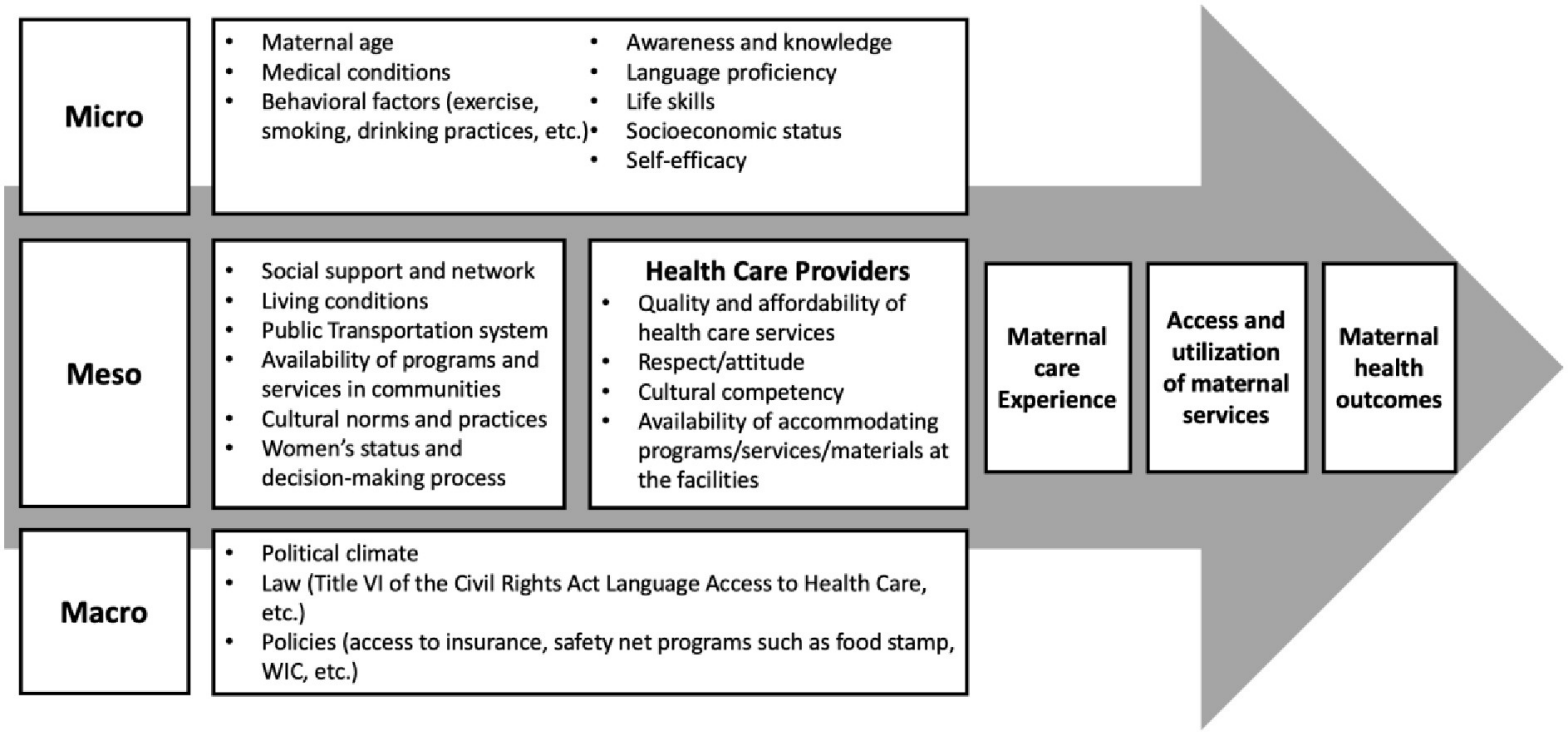
A conceptual framework to explore refugee maternal health.

We propose a conceptual framework that may aid in elucidating the process of refugee women experiencing the maternal healthcare system after they resettle in the United States (Fig 2). The framework incorporates factors that are potentially associated with maternal health outcomes, as informed by other frameworks available [24–27], a scoping review of maternal health among resettled refugee women in the United States [28], and themes and codes gleaned from this study. In this paper, we highlight several factors identified through interviews in each domain: perceptions of hospital and prenatal care, language proficiency, and life skills at the micro-level, cultural norms and practices, social support, and health care provider characteristics at the meso-level, and the healthcare system and access to insurance at the macro-level.

### Micro-level factors

Micro-level factors found in this study include women’s perceptions toward hospital and prenatal care, life skills, and language proficiency. According to the interviews, women who believe the hospital is a place to go only when they are sick, as well as those with limited life skills and language proficiency, are less likely to utilize maternal healthcare services.

#### Perceptions toward hospital and prenatal care

When discussing their maternal healthcare experiences in their home country, some women mentioned that people only sought care when they were sick.

“If they think that their health is good, they are not going (to the prenatal care).

(Interviewee 10, Afghanistan)”

Another Afghan woman, who began her prenatal care visit one and a half months after she had conceived, went back for her second visit in the fifth month and discovered she had lost her baby. She stated that the reason she did not go for the second visit sooner was because her “health was good”.

“The first when I went, I was one and half months pregnant, they tested, and they did an ultrasound for me, but after that no one told me to visit a doctor and my health was good.”

(Interviewee 9, Afghanistan)

#### Life skills

Ten women had no education or had only completed elementary school, and nearly half of them could not read in their native languages. It appeared that some illiterate women, particularly those from rural areas, lacked basic life skills. For instance, they were unfamiliar with maps and had little idea how to use them, which may hinder their ability to navigate their way to a clinic or hospital.

“I know how to take a bus and go somewhere that I know. If I know about the place before. Like, I know how to take a bus to that place, but for places that I don’t know or I haven’t been I can’t because I cannot read the map.”

(Interviewee 9, Afghanistan)

Another woman stated that she had missed two appointments for her newborn baby and two for her other children due to unfamiliarity with the bus system or a lack of knowledge on how to read the map.

“I left home to go to the hospital and use a bus, but on the way, when I saw that the time is late and the bus was coming late, then I came back home and because of the bus, I missed that appointment. Even the doctor called me for a reminder, but I don’t know the bus system.”

(Interviewee 11, Afghanistan)

#### Language proficiency

Some women who could read in their native languages used online translating apps to translate text messages from clinics, but the illiterate appeared to have no idea. Because they could not read or write, they mostly communicated through voice messages with one another. Furthermore, despite their years in the United States, most of the women interviewed could not communicate in English. One woman who has been in the country for more than five years stated that she was still unable to make appointments on her own.

“I still don’t feel able or comfortable plus, you know, when you make an appointment for sickness or something, they ask why do we need to see your son today. You need to be specific and give the exact symptoms or so I don’t feel I am at the level to tell them.”

(Interviewee 16, Syria)

### Meso-level factors

Meso-level factors discussed in this paper include cultural norms and practices, social support provided by various actors around refugee women, as well as health care provider characteristics such as perceived quality and affordability of healthcare services, respect and cultural competency, the availability of accommodating services, and programs such as interpretation services.

### Cultural norms and practices

Several themes concerning cultural norms and practices emerged from the interviews, including a preference for female health care providers and cultural norms and perceptions of family planning and pregnancy.

*Preference towards female health care providers*. Except for one woman, all women preferred female health care providers and were all seen by female health care providers. Sometimes, they said they specifically requested it, and other times health care providers arranged it without asking.

“In our tradition, in our culture, we prefer women to take care of us.”

(Interviewee 4, Syria)

“Yes, she was a female, but it’s not because I requested it, it’s them because they’re so nice. They (health care providers) know that we come from a culture, we care about this stuff.”

(Interviewee 14, Syria)

*Cultural norms about birth control and family planning*. Many women appeared to be open to the idea of birth control, as several women mentioned that they were taking birth control pills or had a device for the purpose. Also, an Iraqi woman mentioned that birth control is culturally accepted.

“It’s better to be on certain family planning than getting pregnant and then if you don’t want the baby, then it’s a problem because abortion is forbidden by (our) religion. But taking care of it (by birth control), if you don’t wanna get pregnant, there are ways that are okay.”

(Interviewee 15, Iraq)

However, the decision seems to be dominated by men or other family members rather than women.

“They (Afghan women) don’t have any plans for, uh, how many children they have, because it’s not a woman’s decision usually, it is a man, mother-in-law, and father-in-law’s decision—how many grandchildren they want and how many children the husband wants.”

(Interviewee 17, Afghanistan)

#### Social support and network

As previously stated, most women had limited English proficiency and could not make a medical appointment on their own, regardless of length of stay in the United States. As a result, they were heavily reliant on social support. Initially, they received support from refugee resettlement agencies for three to seven months based on women’s discourses. When the assistance ends, women are more likely to rely on social support from friends or children if their children are old enough. Those who had relatives already living in the United States were in a better position, but many other refugee women seemed to suffer.

*Refugee resettlement agencies*. For the first few months, refugee resettlement agencies provided primary support, enrolling them into different social services, completing necessary paperwork and training them, making essential medical appointments, taking them to a clinic, and/or arranging transportation as necessary.

“When you first come here you don’t know anything, so they (the refugee resettlement agency) helped us, showing us, or telling us about the insurance, how to go to the doctor, or they took us to the doctor, social security administration office, all this stuff.”

(Interviewee 16, Syria)

Although their assistance is critical and extremely beneficial, particularly during the initial phase of resettlement, the quality of services seemed to vary greatly depending on the organizations and caseworkers, even within the same organizations. Regardless of the organizations or caseworkers assigned to refugee women, they all received administrative support for the paperwork required for insurance or other governmental services. Other support, such as making an appointment or providing training to navigate life in the country, appeared to vary.

“Before, everything was in the hands of the organization, they sent us a car, and they worked on our appointment, but now we cannot do anything. They managed by themselves, but they didn’t train us.”

(Interviewee 8, Afghanistan)

“They (organization B) said that you have to use a bus. You have to know the way. Just two times they helped us. Then they said you have to use a bus and you have to know by yourself.”

(Interviewee 11, Afghanistan)

“If the organization provides a car, we can reach to our appointment, if not, we miss.”

(Interviewee 9, Afghanistan)

Furthermore, although the agencies universally provided administrative assistance for applying for various government programs such as Medicaid and food stamps, some refugee women were unaware of specific coverage or their entitlements.

“I can tell you with confidence, I also was under support of the resettlement agency, nobody described for me. What is Medicaid? How it works? What is in my plan? What plan means? What is primary care, what is urgent care, what is emergency care, when I should go to any of them, no no no information, no enough information.”

(Interviewee 17, Afghanistan)

The participant assumed that one case worker was responsible for too many families and that the organizations did not have a standardized and clear plan for refugees.

“(There should be) enough number of the caseworkers for families because I think the number of case worker was a little, and uh, they worked for lots of families. … I think the caseworker do not spend enough time with family to orient them, and they don’t have really a clear orientation plan for family.”

(Interviewee 17, Afghanistan)

*Friends*. “American friends,” mostly volunteers from local organizations or communities, helped them learn English and make appointments, transported them to clinics, and threw baby showers for women on occasion. Refugee women also helped one another by caring for each other’s children in an emergency, offering them a ride if they knew how to drive, or helping with other important matters.

“I have two friends from Iraq, and they were like, we don’t know how to go, what to do. And one of them was like, take me here, take me there. And I was like, I can help you. So I started teaching them how to drive, I also helped her apply for food stamps.”

(Interviewee 15, Iraq)

*Family/relatives*. When family members or relatives were already present in the resettled region prior to resettlement, the resettlement process appeared to be easier and smoother because their relatives were providing the necessary support.

“I didn’t use public transportation because I have family here. So let’s say my dad or my brothers, whoever is available, even though the hospital will ask—do you need any transportation? I would say no. Thank you. I’m okay.”

(Interviewee 16, Syria)

Relatives were also important sources of support in certain situations, such as after giving birth. Some women reported that their relatives assisted them with housework during their postpartum period.

“For the first couple of days, a relative came and stayed with me at home. She’ll make sure to cook and she’ll come here, make sure to clean the house, make sure to cook. So, yes, the family really supported me until I was able to move freely.”

(Interviewee 12, Syria)

Women who received social support from relatives or family members had very different experiences than those who did not. One woman had her first child after arriving in the United States, and her baby was taken from her for three months until she was reunited with her with the help of a lawyer affiliated with the refugee resettlement agency.

“So as soon as we got here (in the US), I was taken to the hospital to deliver. And when I went home, my husband, like a couple of days later, he started work. They took him to start work. So I was by myself with my first baby, not knowing how to deal with the baby. And the baby was crying. I don’t have this experience. I don’t know how to take care of the baby. Plus I am like under cultural shock, and where we were living is far away. So the neighbors heard the baby crying, they called the police and they thought that I am a neglecting mom or something. So they took my baby from me. So my baby was taken from me because they thought that I was not able to take care of my baby. … It’s so difficult when you don’t have someone to help you. When you first come no one is for you to help you.”

(Interviewee 15, Iraq)

*Support from children*. Women who stayed in the United States for a longer period of time had grown-up children who assisted them in navigating the health-care system.

“My kids started to know English, so they started making the appointments for me.”

(Interviewee 1, Iraq)

“No, I’m still not able to call and make appointments. It’s my daughters (who are making an appointment) … and my daughters or husband, whoever is available is taking me to hospital.

(Interviewee 14, Syria)

#### The characteristics of health care providers

*Perceived quality and affordability of healthcare services*. Overall, women appeared satisfied with the healthcare services they received in the United States in terms of how they treat women, costs, quality of care, and facilities when compared to services provided in their home country. To describe the difference they perceived, two women used the expression “the difference is like the distance between the sky and the earth”.

“The difference (in maternal healthcare services between Iraq and the US) is so big. It’s like the distance between the sky and the earth. The difference, I cannot describe it even, it’s unbelievable. We were surprised by the level of care here. How they take care of the pregnant lady, the baby. Over there, it’s like nothing.”

(Interviewee 15, Iraq)

“There is a lot of difference. Like in Afghanistan, there are no such facilities like here.”

(Interviewee 11, Afghanistan)

*Respect and cultural competency*. All the interviewed women seemed satisfied with the maternal care they received and they felt respected at the healthcare facilities. One woman also stressed that she felt the health care providers were respectful of her needs and culture.

“They were really good. Even though I come from different background, I wear a scarf (Hijab) but that wasn’t like an issue. They were so respectful of my needs and understanding that as like a veiled woman. I had to go through surgery at a certain point. So if by accident, my hair is showing, they will cover my hair. So they didn’t let me feel that I’m a stranger.”

(Interviewee 13, Syria)

*Frequent visits enabled by consistent reminders*. The frequency of prenatal visits was one of the most significant differences the refugee women had noticed in maternal healthcare in the United States. They reported that they were asked to visit health care providers more frequently, and they were reminded to do so, unlike in their home countries.

“Here it’s more consistent. They will call you to remind you—you need to come, we need to check on you. I feel that here it’s almost every month. In Syria, it depends on your financial situation. So if you can afford to go to see the doctor every month, you can go, if you can’t afford it, you don’t go and there in Syria, they’re not gonna call you to remind you about your appointment or anything. If you don’t go see the doctor, that’s it.

(Interviewee 12, Syria)

*Interpretation services*. Interpretation services were provided by health facilities during maternal care visits and delivery. When health care providers called, they asked if they needed interpretation and accommodated their requests by connecting them with interpreters in line. The services were available throughout the delivery process, and one woman who had a miscarriage in the United States reported that the interpreter stayed up all night interpreting the entire procedure. The majority of the women appreciated the interpretation services.

“They had interpreters (over the phone) and I was able to communicate with them in a pretty good way.”

(Interviewee 2, Iraq)

The gender of the interpreters and the quality of interpretation services, however, were sources of discomfort for some women. For some Muslim refugee women, the gender of the interpreters were significant due to their unease in discussing matters pertaining to their intimate body areas with a male interpreter. When requested, the gender of interpreters could be matched, but some women seemed unaware of this accommodation.

“Sometimes because when we talk with your OB (obstetric) doctor about some examination, so it’s difficult to tell them (male interpreters) anything free. So sometimes I feel reluctant to share some of the things.”

(Interviewee 7, Afghanistan)

*Uncomfortable procedure*. When asked if there had been any procedures that had made them uncomfortable, many women stated that there had been none.

“I was okay. And they were very nice and gentle. (They said) Whenever you feel uncomfortable or you are in pain, just raise your hand and let us know. And of course, I was like cooperating and I was okay with all these tests and procedures, because they’re doing this for my benefit and benefit of the baby. So I was always like cooperating and not minding or all this.”

(Interviewee 15, Iraq)

A few women, however, mentioned about cervical examination as an uncomfortable procedure. One woman even refused to have a cervical examination.

“Just once when they wanted to check my bottom to know that my pregnant period will come or not. And I refused that and said I know I have a baby. There is no need to check my body. And they said, okay, if you don’t want, we will not do.”

(Interviewee 6, Afghanistan)

Another woman mentioned the cultural norms concerning the cervical examination especially for unmarried women.

“In our countries, this, this doesn’t happen. It’s like, you are not approved until you are married.”

(Interviewee 4, Syria)

*Communicating negative issues*. One intriguing theme that was identified in several interviews was the difference in how negative issues are addressed and communicated in different cultures.

“One thing I noticed here that is not in Syria is that here let’s say you are about to go into surgery. And they will explain to you what might happen. Like negatively, let’s say, what are the side effects? So this was affecting me a lot. I’ll be really prepared, but then when they talk to me about all the potential, so I’ll be stressed, scared. This doesn’t happen in Syria because, well, it’s a culture and religious thing a little bit because there, like we say, or we believe that we’ll do our best and the rest is in God’s hands, you know? So here, no, they make sure they’re explaining to you everything because they don’t wanna be responsible for anything in case something happens to you. In Syria, it will be God’s fault in Syria, it’s a little bit the opposite. The doctor will be joking with you and reassuring you that everything will be okay. And here it’s a little bit different. It’s the opposite, but I’m getting used to it. Yeah, definitely, believing that everything is as God wishes it to happen.”

(Interviewee 14, Syria)

“In Afghanistan, people are more indirect when they’re saying those kinds of things. They talk to families like a mom instead of the women. (When I hear those negative things) Sometimes I feel so bad. I’m here alone, I am new as a refugee. And they told me anything… I think it is the negative part.”

(Interviewee 7, Afghanistan)

### Macro-level

The political climate, laws, and policies all have an impact on refugee women’s experiences, as well as their access to and utilization of maternal care services through different pathways. In this study, the healthcare system in the country and access to insurance were identified.

#### Complex healthcare system in the United States

Some women appeared to be challenged by the complex healthcare system in the country and their unfamiliarity with it. No appointments were required in Afghanistan, for example, and pharmacies were located in health facilities. In the United States, on the other hand, women must first determine whether a specific clinic accepts their insurance and accepts new patients. Then, they must make a phone call to schedule an appointment using various automated voice instructions, visit healthcare facilities, and, if necessary, travel to a pharmacy in a different location. Errors were common, so they had to alternate between a clinic and a pharmacy. Even for those born and raised in the United States, navigating the healthcare system can be challenging. It is often fragmented and difficult to comprehend, resulting in "confusion, frustration, distress, anger, and loss of control" [25]. One woman stated that one year after resettlement, she and her children still do not have a primary care physician, and they go to urgent care as needed.

“It’s one year I’m in the USA. And still, I don’t have a primary care provider, because I have to call to one million of the primary care providers and ask, “Can you please accept new patient?” And then they say, at this time we cannot accept any patient. I have at least a hundred of the providers, which is under the network of my insurance. So it has to be insurance network, which is not easy for Afghan women to understand, but for me, because I kind of speak English, so I understand this is the process. So I call different primary care providers, which was listed in the website of Medicaid, as they are in the network, and all of them do not accept me yet. It’s one year, and I’m waiting. So anytime I have a problem, I have to go to an urgent here.”

(Interviewee 17, Afghanistan)

One refugee woman went to an urgent care clinic just to test her pregnancy due to unfamiliarity with the healthcare system.

“Originally, we didn’t have a lot of experience, so my husband told me maybe we can go there (an urgent care clinic) and then we can do a test and then they will tell us where to go.”

(Interviewee 2, Iraq)

#### Access to insurance

All of the women had Medicaid insurance and stated that they did not pay out-of-pocket expenses for maternal care services. However, their knowledge of other services covered by their insurance, such as medical transportation that provides free ground transportation to receive medically necessary covered services, varied significantly. One woman met an American family at an apartment event who informed her of the services and has used them since, but the majority of the others were unaware of the service.

“We were not aware of it (medical transportation). Yeah, we struggled a lot.”

(Interviewee 15, Iraq)

Another woman stated that other refugees do did know what they were entitled to or what their insurance coverage was, and as a result, they sometimes refused to seek care.

“A woman in my neighborhood gave birth a few months ago, and she suffered from different kind of problems. Sometimes she come to me because I’m a midwife, and I say, you have to go to the gynecologist and then she says, No, no, my husband says you’re not allowed to go to the doctor too much, because if you go more than, for example, two or three times and deducted, then we have to pay a co-pay we don’t have money, so I’m not allowed.”

(Interviewee 17, Afghanistan)

#### Maternal mental health and mental health screening

One of the interview questions was to inquire if they had received any depression or mental health screening as part of their maternal healthcare. Most women reported that their health care providers asked them if they had depressive symptoms and their responses were no.

“So when I used to do follow up with the clinic, they would ask me questions, how was your mental health, is everything okay, anyone hurting you, and stuff like that, and no, I was fine.”

(Interviewee 1, Iraq)

Although no women, with the exception of one, explicitly stated that they had been diagnosed with depression, some appeared to have multiple stressors in their lives. One woman expressed her sorrow for family members she had left behind in their home country, another had miscarriages, and the other had a family member murdered in their country of origin.

“I was really depressed (about my miscarriage) because I was so excited that it was twins and I was hoping it was a boy also, because I wanted a boy. I wanted a brother for my daughter. So losing, losing two… But yeah, so I had to miscarry them… Because they were like dying inside me.”

(Interviewee 15, Iraq)

“So I’m always sad. My daughter is far away. I cannot do anything for her. And I am so emotional. And she’s the oldest daughter. And my mom is in Aleppo and she lives by herself also. And she has cancer also and she’s by herself, no one there to take care of her. So I went through lots of hard times and sad times. Inside me, I’m not so happy because I’m away from all those (my mom and my daughter).”

(Interviewee 14, Syria)

“Because I have lost my brother-in-law in Afghanistan. Taliban killed him by shooting and I was not able to speak or go anywhere.”

(Interviewee 11, Afghanistan)

## Discussion

This research identified multiple themes that may enhance understanding of the maternal care experience, access to, and utilization of maternal care services among Muslim refugee women resettled in the United States. In general, Muslim refugee women understood the importance of maternal healthcare but had been unable to access healthcare services due to geographical accessibility, costs, or cultural beliefs that hospitals are only for the sick in their home country. In the United States, most of the women interviewed had access to and used maternal healthcare services. Perceived quality, no out-of-pocket costs, respect and cultural competency of health care providers, constant reminders from health care providers, the perceived importance of receiving maternal care, scheduling the next appointment in advance during the visit, and interpretation services provided at healthcare facilities all aided in this. They generally felt respected by health care providers and seemed satisfied with the services and facilities in the United States because they were perceived to be better than those in their home countries.

Nonetheless, regardless of the length of stay in the United States, many women have limited English proficiency and are unable to make medical appointments on their own, which is consistent with previous research [29]. Thus, the level of access and utilization varied according to the level of support and language proficiency, as well as educational level, given the complexity of the health system in the country. The quality of services provided by refugee resettlement organizations appeared to vary significantly as well. Even though the interviewed refugee women have universal access to insurance, their level of awareness and knowledge about coverage and entitlement varied significantly, owing in part to a lack of standardized training as part of the resettlement process. In addition, many of the women interviewed had multiple risk factors, such as chronic diseases, high blood pressure, diabetes, or a history of multiple miscarriages. They were also subjected to a variety of stressors and difficulties in life.

### Implications for practice and future research

Access to and utilization of maternal healthcare services was highly variable. First-time mothers or mothers with young children, uneducated, illiterate women lacking basic life skills, and those without families or relatives appeared to be the most vulnerable, with limited access to maternal and general healthcare. As a result, more tailored programs for the most vulnerable groups of refugee women are needed. Furthermore, while refugee resettlement agencies provide much-needed assistance in applying for various programs, including insurance, more standardized training should be provided to help refugee women navigate the complex healthcare system in the United States, increase their knowledge of their entitlements and community resources, and empower women to navigate the complex healthcare system. Furthermore, the training’s content and pace should be tailored to the level of education and literacy. In addition, due to limited health literacy, English proficiency, and unfamiliarity with the healthcare system, refugees are likely to have difficulty navigating the healthcare system even after the support from refugee resettlement agencies ends. A long-term community-based health navigation intervention could be one of the potential programs that can help refugee women get better access to healthcare [29].

There could also be more support during the postpartum period. According to one empirical study, a postnatal nurse home-visiting intervention could reduce infant medical visits and anxiety among mothers while also enhancing positive parenting behaviors [30]. Given the vulnerability of refugee mothers and their limited social support and network, postpartum refugee women could benefit from programs such as this.

Despite the fact that many health care providers appear to provide respectful and culturally appropriate care, communication, particularly communication of negative issues, could be improved given women’s unique experiences and circumstances. This could be aided by culturally appropriate communication that incorporates their cultures.

This study also identifies several areas for further investigation. Although not directly related to maternal healthcare, many interviewed women and their families appeared to suffer from chronic diseases. However, the prevalence of chronic diseases in this population has yet to be investigated. After controlling for sociodemographic factors, one study in the United States concluded that refugees were more likely to have chronic conditions than other groups of immigrants [31]. As chronic diseases are associated with high levels of stressors in life [32], potential causes and pathways could be investigated further in the long term.

Another area that requires more attention is mental health. Refugees are likely to face a variety of stressors, including trauma experienced prior to resettlement, economic stability, discrimination, and cultural differences [33, 34]. However, mental health is viewed differently in various countries and cultures, and it is either not recognized or considered taboo in many. Although few women reported having issues with maternal depression during the interviews, they did report a variety of stressors, including being cut off from their traditional network and social support. During an interview, an Afghan interpreter who was interpreting the question said it was "the hardest question".

“It is the hardest question. Because most of women or every person in Afghanistan do not know exactly what it is (depression).”

(Interpreter, Afghanistan)

Further investigation is warranted to explore effective approaches for addressing mental health concerns, particularly among individuals from diverse cultural backgrounds, wherein mental health issues might be deemed sensitive and linked to significant social stigma.

Lastly, it might be interesting to investigate how different policies, laws, and community resources could shape the experiences of women and influence the utilization of maternal healthcare services. The experience of refugee women in a host country that lacks interpretation services or culturally appropriate care, as depicted above, would be vastly different from that of those who have those accommodations.

### Limitations and strengths

This is the first study to use a comprehensive framework to investigate maternal health among Muslim refugee women resettled in the United States. The framework could be useful not only for studying refugee populations but also for studying immigrants who must navigate an unfamiliar healthcare system. However, the study has several limitations. Because of the recent upheaval in Afghanistan, most of the new arrivals were Afghans, while there were fewer new arrivals from other countries, such as Iraq and Syria. This also affected recruitment, with more Afghans being recruited compared to other Muslim-majority countries. This trend mirrors the recent influx of Afghan refugees, whose numbers have doubled compared to those from Iraq and Syria. The results need to be interpreted considering these circumstances. Furthermore, because the recruitment was initially done through contacts provided by refugee agencies, selection bias may be present. However, later in the recruitment process, snowball sampling was used, and women were introducing other women they met at various venues.

## Conclusions

Understanding and promoting health necessitates both individualistic and structuralist approaches, as human health is both an individual and a social issue [18]. In this study, maternal care experiences, as well as access to and utilization of maternal healthcare services, were investigated at micro, meso, and macro-levels. Understanding refugee maternal health requires a more nuanced understanding, given the heterogeneity of the population. Even among women from the same country, their experiences varied greatly depending on multiple factors at individual and structural levels. Addressing the issue of health inequity requires delving into and unearthing the complexities hidden beneath the seemingly homogeneous group known as refugee women, as well as meeting needs through more tailored support. As one woman put it, “no matter what, life goes on. Good or bad, it will go on.” And it is our responsibility to shape how their lives unfold in this country.
